# 

**Published:** 1879-06

**Authors:** 


### CONTENTS:

ARTICLE I.—Treatment of the Dental Pulp. By Arthur M. Rice, D. D. S. .......... 49
ARTICLE II.—The Origin, Progress and Present Condition of Dental Science. By Geo. H. Chewning, D. D. S. .......... 56
ARTICLE III.—False Diplomas. By A. M. I. Hein, D. D. S. .......... 65
ARTICLE IV.—On General and Local Acidity. By John C. Peters, M. D .......... 68
ARTICLE V.—Chloroform in Convulsions of Infants and Children. By J. D. Blake, M. D. 80
ARTICLE VI.—The Goodyear Company's Patent .......... 85
ARTICLE VII.—Dental Associations—National Dental Association .......... 87
South Carolina Dental Association .......... 88
Georgia State Dental Society .......... 89

### EDITORIAL, ETC.

The National Dental Association .......... 89
Maryland & D. C. Dental Association Proceedings .......... 90

### OBITUARY.

Alfred J. Brown, Sr. D. D. S. .......... 92

Bryant S. Traywick, D. D. S. .......... 92

### BIBLIOGRAPHICAL.

Transactions of the American Dental Association .......... 93
Rhymes of Science .......... 93

### MONTHLY SUMMARY.

Rapid Anæsthesia .......... 93

Mal-Practice Case .......... 94

Chewing Gum .......... 95

A Remarkable Old Man .......... 95

Chloroform Accidents .......... 96

How to Care for the Eyes .......... 96

Action of Substances on the Teeth .......... 96

